# A case report of a patient with primary familial brain calcification with a *PDGFRB* genetic variant

**DOI:** 10.3389/fneur.2023.1235909

**Published:** 2023-09-14

**Authors:** Jamal Al Ali, Jessica Yang, Matthew S. Phillips, Joseph Fink, James Mastrianni, Kaitlin Seibert

**Affiliations:** ^1^Department of Neurology, University of Chicago, Chicago, IL, United States; ^2^Department of Psychiatry and Behavioral Neuroscience, University of Chicago, Chicago, IL, United States

**Keywords:** Fahr’s disease, missense variant, PDGFRB gene variant, primary familial brain calcification (PFBC), tyrosine kinase

## Abstract

Fahr’s disease, or primary familial brain calcification (PFBC), is a rare genetic neurologic disease characterized by abnormal calcification of the basal ganglia, subcortical white matter and cerebellum. Common clinical features include parkinsonism, neuropsychiatric symptoms, and cognitive decline. Genes implicated in Fahr’s disease include *PDGFB*, *PDGFRB*, *SLC20A2*, *XPR1*, *MYORG*, and *JAM2*. We present the case of a 51-year-old woman who developed subacute cognitive and behavioral changes primarily affecting frontal-subcortical pathways and parkinsonism in association with extensive bilateral calcifications within the basal ganglia, subcortical white matter, and cerebellum on neuroimaging. Relevant family history included a paternal aunt with parkinsonism at age 50. Normal parathyroid hormone and calcium levels in the patient’s serum ruled out hypoparathyroidism or pseudohypoparathyroidism as causes for the intracranial calcifications. Genetic panel sequencing revealed a variant of unknown significance in the *PDGFRB* gene resulting in a p.Arg919Gln substitution in the tyrosine kinase domain of PDGFRB protein. To our knowledge this is the first report of a p.Arg919Gln variant in the *PDGFRB* gene associated with PFBC. Although co-segregation studies were not possible in this family, the location of the variant is within the tyrosine kinase domain of PDGFRB and pathogenicity calculators predict it is likely to be pathogenic. This report adds to the list of genetic variants that warrant functional analysis and could underlie the development of PFBC, which may help to further our understanding of its pathogenesis and the development of targeted therapies for this disorder.

## Introduction

Primary familial brain calcification (PFBC), also known as Fahr’s Disease, is a genetically and phenotypically heterogenous neurodegenerative disorder (1–3). Clinically, patients with PFBC experience a variable combination of neuropsychiatric (4–10) and motor symptoms (2, 11, 12), including parkinsonism, dystonia, seizures, ataxia, chorea, dementia, psychosis, and frontal-subcortical cognitive dysfunction. Radiologically, abnormal calcification is present within the bilateral basal ganglia, but also the subcortical white matter, cerebellum, thalamus, and brainstem (1–3).

**Figure 1:**
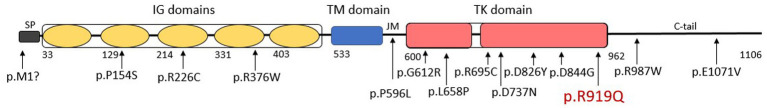
A schematic representation of Platelet Derived Growth Factor Receptor beta (PDGFRB) protein including a signal peptide (SP), five extracellular Ig-like (IG) domains, a transmembrane (TM) domain, a juxtamembrane domain (JM), an intracellular split tyrosine kinase (TK) domain, and a C-terminal tail (C-tail). Previously reported variants affecting the PDGFRB protein are presented according to their approximate position in the protein amino-acid chain. The patient’s p.R919Q variant is shown in red.

Six genes contribute to the genetic heterogeneity of PFBC, four of which follow autosomal dominant inheritance: *PDGFB*, *PDGFRB*, *SLC20A2*, and *XPR1* (3, 12–15) and two are autosomal recessive: *MYORG*, and *JAM2*; (12, 16, 17). We describe a case of a 51-year-old woman with cognitive, behavioral, and radiographic features of Fahr’s disease who harbored a variant (Rs14571770) (18) of the *PDGFRB* gene (Platelet Derived Growth Factor Receptor beta). The transition c.2756G>A resulted in a glutamine substitution of arginine (p.Arg919Gln) in exon 20, within the tyrosine kinase domain of the PDGFRB protein (Figure 1) (19).

Three gene panels were used to screen for genetic variants in a total of 70 genes implicated in neurodegeneration and PFBC (Supplementary material). Genes implicated in PFBC were *SLC20A2, PDGFB, PDGFRB and XPR1.* At the time of testing, available panels did not include sequencing for the two autosomal recessive genes *MYORG* and *JAM2. A*ll 3 panels used next generation sequencing of the exons; and analyzed the sequences for missense variants, insertions, deletions, and copy number variations.

Detection of this variant in *PDGRFB* assisted in diagnosis and management of this patient, emphasizing the importance of genetic testing in patients with neuropsychiatric symptoms, parkinsonism and neuroimaging characteristics suggestive of PFBC.

## Case presentation

**Figure 2:**
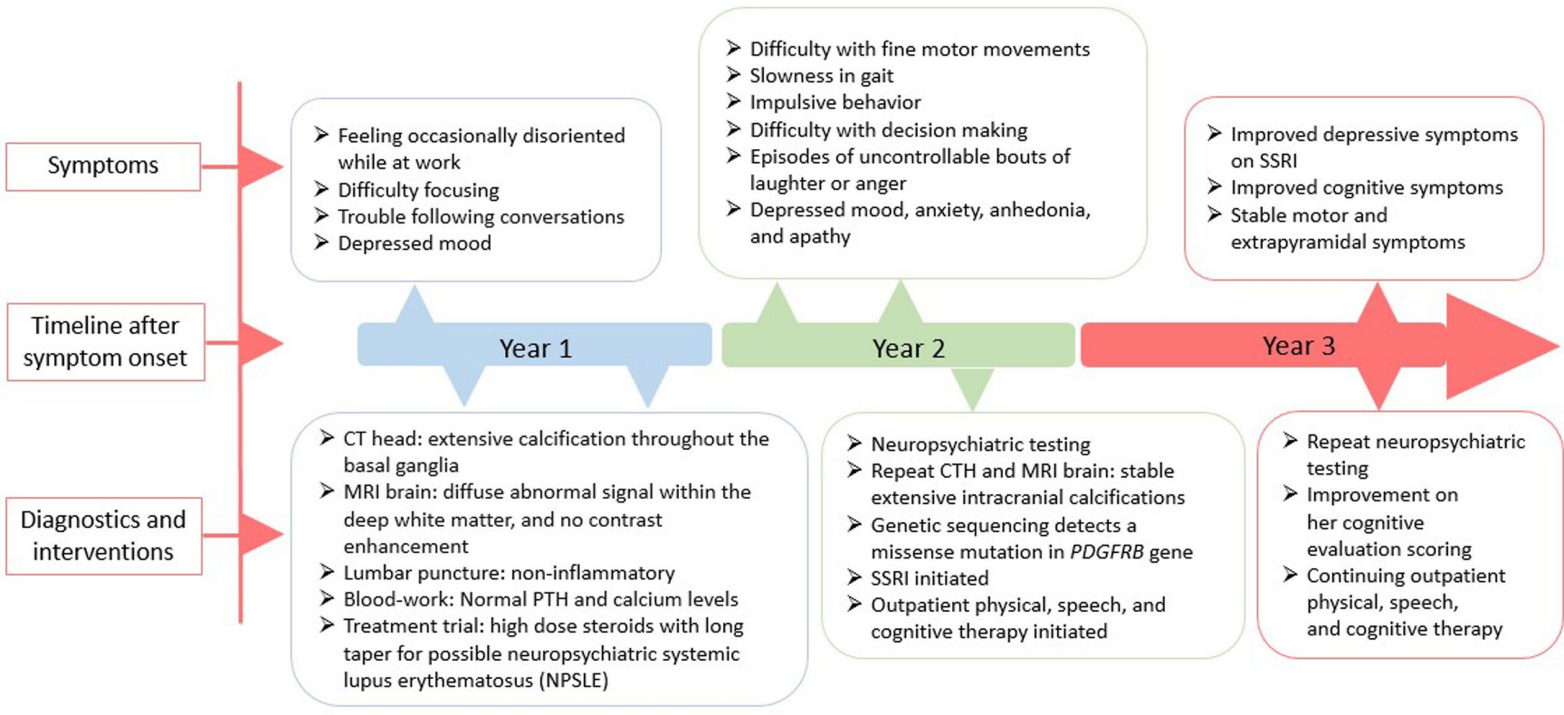
A schematic timeline of the patient’s symptoms, diagnostic workup, and interventions, presented in years after presentation.

A 51-year-old Filipina woman with a history of hypertension and systemic lupus erythematosus (SLE) presented with subacute cognitive changes over the course of 4 weeks. The patient reported feeling occasionally disoriented at work, with difficulty concentrating, and depressed, which were noticed by her family and coworkers. Additional symptoms noted by her family included dysarthria, dysphagia, gait instability, and trouble following conversations. She had no personal psychiatric or neurologic history. Family history was significant for several family members with rheumatoid arthritis and SLE, and a paternal aunt with parkinsonism at the age of 50, for whom an autopsy was not done. The patient’s father died at age 74 from small cell lung carcinoma and her mother died at age 70 from cardiac arrest. Neither parent had parkinsonian or cognitive symptoms, although a paternal aunt was diagnosed with Parkinson’s Disease at age 50. Evaluation at a local hospital included a computerized tomography (CT) scan of the head, which showed extensive hyperintensity throughout the basal ganglia, cerebellum, central pons, and periventricular subcortical white matter. Initial magnetic resonance imaging (MRI) of the brain with gadolinium showed diffuse abnormal susceptibility signal within the deep white matter in the cerebellar and cerebral hemispheres, relatively minimal abnormal fluid-attenuated inversion recovery (FLAIR) signal, and no contrast enhancement. Lumbar puncture was performed to assess for inflammation in the setting of possible neuropsychiatric SLE (NPSLE); cerebrospinal fluid (CSF) testing was non-inflammatory, with white blood cell count 3 (nL = 0–5 cells/mm^3^), Glucose 76 (nL = 40–70 mg/dL), Protein 35 (nL = 15–45 mg/dL), and negative results for Gram stain and culture, West Nile virus, Herpes Simplex Virus (HSV), Measles, Mumps, Varicella Zoster Virus (VZV), and Coccidioidomycosis. Serum studies showed a positive Antinuclear Antibody (ANA) titer of: 1:2,560 (nL = <1:40) speckled, negative double stranded deoxyribonucleic acid (dsDNA) Antibody < 1 (nL = <4 iU/mL), normal complement component 3 (C3): 123 (87–200 mg/dL), and normal complement component 4 (C4): 32 (19–52 mg/dL). Based on a concern for NPSLE, she was treated with intravenous methylprednisolone 1 g/kg/day for 3 days followed by a prolonged oral prednisone taper. For depressive symptoms, she was prescribed citalopram 20 mg. A neuropsychological evaluation was ordered, and she was referred to a tertiary center for further diagnosis and management. A timeline of the patient’s symptoms, diagnostic workup, and interventions is represented in Figure 2.

The patient underwent the neuropsychological evaluation but did not follow up in clinic until 15 months later. At that time, she presented with concerns of symptom progression. She reported trouble with fine movements, especially writing, slowing of her gait, and falling. Her dysarthria worsened and she developed dysphonia and dysphagia. Behavioral changes included new impulsivity and episodes of uncontrollable bouts of laughter or anger. On neurological examination, the patient was alert and oriented to person, place, and time, her speech was slow, deliberate and aprosodic. She exhibited normal naming, comprehension, and repetition, with no paraphasic errors. Ideomotor apraxia was demonstrated in both hands. She scored 16 on a Montreal Cognitive Assessment (MoCA), missing points for Trails B (−1), cube copy (−1), clock draw (−2), animal naming (−1), backward digit repetition (−1), serial seven subtraction (−2), sentence repetition (−2), phonemic fluency (−1), abstraction (−2), and delayed recall of one of five words (−1), although she retrieved the word with a semantic cue. Cranial nerve evaluation demonstrated oculomotor apraxia with hypermetric saccades and impaired smooth pursuit, hypophonia, and hypokinetic dysarthria. The motor exam showed paratonic upper extremities, bradykinesia and diffuse hyperreflexia. Plantar reflexes were flexor bilaterally. Primitive reflexes of grasp., glabellar, palmomental, and snout were present. Dysmetria was present bilaterally, although more prominent on the left. Her posture was narrow-based, upright, but with reduced stride and absent arm swing on the left. Sensory exam was normal.

**Figure 3:**
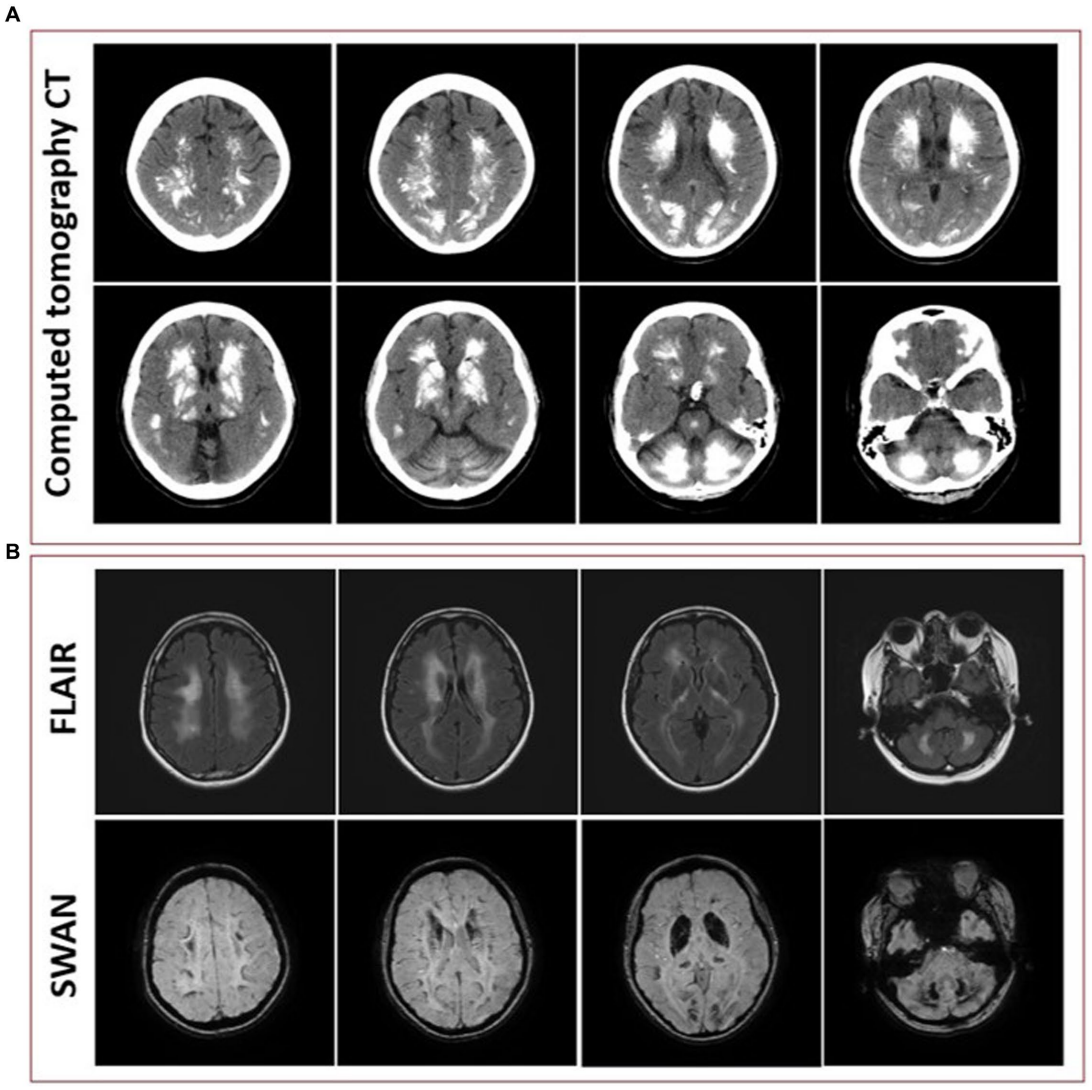
**(A)** Representative axial cuts from the patient’s computed tomography (CT) scan 15 months after symptom onset, showing diffuse hyperdense foci favored to represent extensive calcifications throughout the bilateral corona radiata, basal ganglia, cerebellar hemispheres, and midbrain. **(B)** Representative axial cuts from the patient’s magnetic resonance imaging (MRI) 15 months after symptom onset, fluid-attenuated inversion recovery (FLAIR) sequence showing high T2 signal in the bilateral cerebral, brainstem, and cerebellar deep gray nuclei and white matter and susceptibility-weighted angiography (SWAN) sequence showing extensive susceptibility effect.

Initial blood work was ordered to exclude metabolic etiologies that might lead to brain calcifications, including parathyroid hormone, calcium, magnesium and phosphate, all of which were normal. A repeat CT scan of the head showed diffuse hyperdense foci favored to represent extensive calcifications throughout the bilateral corona radiata, basal ganglia, cerebellar hemispheres, and midbrain (Figure 3A), comparable to the patient’s initial neuroimaging 15 months prior. A repeat MRI brain without contrast showed extensive susceptibility effect and high T1 and T2 signal in the bilateral cerebral, brainstem, and cerebellar deep gray nuclei and white matter (Figure 3B).

The neuropsychological evaluation (Table 1) done 15 months prior revealed prominent impairment in attention and processing speed that contributed to variably impaired new learning and memory. Impairments were also evident with problem solving, speeded verbal fluency and naming tasks. On a self-report measure of depression (Beck Depression Inventory, 2nd Edition), she endorsed elevated level of depressive symptoms, with feelings of sadness, thoughts of suicide, anhedonia, irritability, and significant fatigue. On a self-report measure of behavioral symptomatology associated with frontal networks functioning (Frontal Systems Behavior Scale), when compared with that at the time of initial presentation, 2 months prior to the neuropsychological evaluation, she endorsed a decrease in measures of apathy, disinhibition, and executive dysfunction, although her partner endorsed no significant changes in these symptoms. A repeat neuropsychological evaluation, approximately one and a half years after the initial evaluation (Table 1), was generally consistent with the findings described in the previous evaluation, although the patient demonstrated some improvement in measures of immediate and delayed memory and recognition; increased difficulty on select measures of visual memory and psychomotor processing speed were also noted (Table 1). She was switched from citalopram 20 mg to sertraline 50 mg daily due to her unresolved depressive symptoms, and was provided physical, speech, and cognitive therapy.

**Table 1:** Neuropsychological test data comparing 2019 evaluation with 2021 evaluation.

		2019 evaluation	2021 evaluation
Domain	Measure	*z*-scores	*z*-scores
Estimated premorbid function	Word reading	0.90	0.75
Attention/working memory	WAIS-IV WMI^1^	−0.55	−0.95
	Digit span	−0.67	−0.67
	Arithmetic	−2.00	−1.00
	WAIS-IV PSI^2^	−1.75	−1.60
	Symbol search	−2.33	−1.67
	Coding	−1.00	−1.33
	Stroop word	−1.50	−3.00
	Stroop color	−1.60	−2.10
	Trails A	−2.40	−2.40
Memory	CVLT-II^3^		
	Total	−1.20	−0.70
	Short delay free recall	−1.50	−1.00
	Short delay cued recall	−3.50	−2.00
	Long delay free recall	−2.00	−1.00
	Long delay cued recall	−2.50	0.00
	RCFT^4^		
	Immediate recall	0.50	−0.40
	Delayed recall	0.50	−1.30
	Recognition trial	−2.95	−2.00
	WMS-IV^5^		
	Logical memory I	−1.67	−0.67
	Logical memory II	−1.00	0.00
	Recognition trial	36%–50%tile	51%–75%tile
Language	Naming	−3.00	−2.67
	Fluency		
	Phonemic	−1.33	−0.33
	Semantic	−1.75	−1.67
Executive functioning	Stroop color-word	−1.60	−1.80
	WCST^6^		
	Categories	6%–10%tile	11%–16%tile
	Errors	−1.60	−1.70
	Perseverative Responses	−1.50	−0.90
	Trails B	−1.90	−2.30
Motor functioning	Grooved pegboard		
	Dominant hand	−2.70	−2.20
	Non-dominant hand	−2.80	−2.00
	Grip strength		
	Dominant hand	−1.90	−1.90
	Non-dominant hand	−1.30	−1.40
Behavioral functioning	FrSBe^7^ (self)	Before/after *(T-score)*	Before/after *(T-score)*
	Apathy	78/58	91/95
	Disinhibition	83/64	75/63
	Executive dysfunction	61/45	96/57
	Total	79/57	95/74
	FrSBe (informant)		
	Apathy	44/51	47/97
	Disinhibition	50/52	48/54
	Executive dysfunction	50/54	46/69
	Total	48/53	47/76

1. Wechsler Adult Intelligence Scale, 4th Edition Working Memory Index; 2. Wechsler Adult Intelligence Scale, 4th Edition Processing Speed Index; 3. California Verbal Learning Test, 2nd Edition; 4. Rey-Osterrieth Complex Figure Test; 5. Wechsler Memory Scale, 4th Edition; 6. Wisconsin Card Sorting Test; 7. Frontal Systems Behavior Scale.

Based on the neuropsychiatric findings, clinical parkinsonism, basal ganglia calcifications, and family history of parkinsonism, the suspicion for PFBC was high which prompted us to search for a possible genetic cause, using a directed approach that focused on genes implicated in PFBC. We detected a c.2756G>A change in the *PDGFRB* gene, which results in a glutamine (Gln) substitution of arginine (Arg) at residue 919 (Figure 1).

## Discussion

We report here the clinical, neuroimaging and neuropsychological features of 51-year-old Filipina woman with PFBC who was found to harbor a missense variant in the *PDGFRB* gene. Using next generation genetic sequencing, we detected a c.2756G>A, p.Arg919Gln substitution in the tyrosine kinase domain of PDGFRB protein (19). Using available computational models, the variant is predicted to be “probably damaging” (PolyPen: 0.981), “deleterious” (SIFT: 0.03), “likely deleterious” (CADD: 32), and “damaging” (MetaLR: 0.56). This variant [NM_002609.4, ENST00000261799.4, chr5: 149499072 (GRCh37/hg19)] is reported in dbSNP (Rs145717708, http://www.ncbi.nlm.nih.gov/snp/) (18), and was found in 33 individuals in gnomAD (SNV 5-149,499,072-C-T, https://gnomad.broadinstitute.org) (20), with a low minor allele frequency of 0.0001202 in the general population and 0.001595 (>0.1%) in the “Other East Asian” population, but has never been reported in association with PFBC or any other pathology. Following the ACMG criteria for scoring genetic variants (21), we would classify this as a variant of unknown significance (VUS) because it satisfies contradictory criteria for being a benign (BS1, the allele frequency is greater than expected for the disorder) and a pathogenic variant (PP3, the variant is located in a well-established functional domain and that multiple lines of computational evidence support a deleterious effect on the protein).

*PDGFRB* gene is known for its pleiotropism, making it central to different molecular systems and implicated in a diverse array of neurological syndromes including infantile myofibromatosis, Kosaki/overgrowth syndrome, Penttitnen syndrome, Sporadic Port-Wine Stain, Moyamoya syndrome, Cornelia de Lange syndrome and PFBC (19). The PDGFRB protein is a widely expressed pericyte marker (22), integral in maintaining the blood–brain barrier (BBB). Dysfunction within the BBB can lead to deposition of aberrant materials in the brain, such as the calcifications seen in PFBC (8).

Among the 13 variants in *PDGFRB* that are reported to be associated with PFBC (Figure 1) (8, 23–29), six are missense variants lie within the tyrosine-kinase domain between exons 13 and 20 (8, 24, 26–29). In cell-based experiments, two missense variants affecting the tyrosine-kinase domain (p.L658P, p.R695C) were shown to directly interfere with PDGFRB autophosphorylation, leading to defective downstream signaling (27, 30, 31). A recent study showed that 4 of the 6 known missense variants in the tyrosine-kinase domain (p.G612R, p.L658P, p.D826Y, p.D844G) resulted in complete loss of tyrosine-kinase activity (29), one variant (p.R695C) had a partial effect on receptor autophosphorylation, and one variant (p.D737N) did not lead to any significant functional defect. The p.R919Q variant we present here warrants further study to investigate its functional effect on the tyrosine-kinase activity of PDGFRB protein.

In a recent systematic review on phenotype–genotype relationships of 516 patients with PFBC, 26 (5%) from 9 families were reported to carry PDGFRB variants, eight of which carried a unique missense variant (12). All 26 carriers had calcification of the basal ganglia and 12 of the 26 variant carriers (46%) were clinically affected. As in the case of our patient, calcifications in other affected areas including thalamus, cerebellum, and white matter were commonly found in symptomatic carriers (12). The median age at onset of PFBC in a PDGFRB carrier in this series was 48 years (range 11–54) (12), which is consistent with our patient who developed symptoms at age 51. The most common motor signs reported included parkinsonism and bradykinesia (17% each), and the most frequent nonmotor signs were headache (33%) and cognitive deficits (25%) (12). In our case, the patient’s predominant symptoms were neuropsychological (cognitive, behavioral, and psychiatric) with only mild motor manifestations (bradykinesia and dysarthria).

The clinical work up of brain calcifications include ruling out an endocrinological source of abnormal calcium homeostasis. We confirmed normal serum parathyroid hormone and calcium levels in our patient, ruling out hypoparathyroidism or pseudohypoparathyroidism as causes. Our patient’s history of SLE also raised concerns for NPSLE. NPSLE is known to present with multiple neuropsychological symptoms including acute confusional states, cognitive, anxiety, and mood disorders; however, less than 1% of patients present with motor symptoms and the diagnosis remains largely a diagnosis of exclusion (32). In our patient, the presence of parkinsonism on exam and the extensive intracranial calcification supports the diagnosis of PFBC over NPSLE. Other adult-onset neurodegenerative conditions with intracranial calcifications include spinocerebellar ataxia type 20 (SCA20) which is associated with pronounced cerebellar calcifications affecting the dentate nucleus without involvement of the basal ganglia; polycystic lipo-membranous osteo-dysplasia (PLOSL) characterized by fractures, frontal lobe syndrome, and progressive dementia beginning in the fourth decade, with bilateral calcifications of the basal ganglia, most often in the putamina; and dystonia, parkinsonism, hypermanganesemia, polycythemia, and chronic liver disease, which is a movement disorder resulting from manganese accumulation in the basal ganglia. This disease results from biallelic loss-of-function variants in SLC30A10 and basal ganglia calcifications may mimic those seen in individuals with PFBC (3).

Several case reports and reviews have explored the neuropsychological profiles of individuals with PFBC (4, 5, 7, 33–35). Psychiatric manifestations including mood disorders and psychotic symptoms are frequently present. Behavioral problems including apathy, disinhibition, aggressiveness, and impulse control disorders are reported, and the cognitive impairment that is describe ranges from mild memory and attention deficits to dementia with a frontal-subcortical profile (5, 36, 37).

As evidenced by the neuropsychological evaluations, our patient had findings of cognitive (impaired attention, delayed processing speed, and executive dysfunction), behavioral (apathy and disinhibition) and psychiatric manifestations (depression and irritability). These findings can be attributed to a dysfunction of the frontal-subcortical circuits including the anterior cingulate, the dorsolateral prefrontal, and the lateral orbitofrontal circuits. According to the Rate Model developed in the late 1980s and early 1990s (38, 39), the basal ganglia are responsible for the execution and maintenance of both motor and cognitive functions (40). Impaired executive function, apathy, and impulsivity, all of which were present in our patient, are likely explained by disturbances in the anterior cingulate and dorsolateral prefrontal circuits that are known to regulate these functions (36, 41, 42). Additionally, mood disorders including depression, also present in our patient, can be attributed to dysfunction in the lateral orbitofrontal circuit (36, 41, 42).

This report demonstrates the importance of genetic sequencing in patients with progressive neuropsychiatric disease and extensive basal ganglia calcification that suggests PFBC. Uncovering the full genetic spectrum in patients with PFBC can contribute to further understanding of disease pathogenesis and may be integral in developing targeted molecular and genetic therapies. Without targeted therapies, the treatment remains supportive with the help of a multidisciplinary team including a neurologist, psychiatrist, psychotherapist, physical therapist, and cognitive and speech therapist. The limitations of our study include the inability to perform co-segregation studies and genetic analysis of parents’ samples, and the inability to sequence the two autosomal recessive genes *MYORG* and *JAM2.* Future studies are warranted to investigate the variant’s functional effect on the tyrosine-kinase activity of PDGFRB protein.

## Patient’s perspective

We thank the patient and her family for allowing us to discuss her clinical course and genetic findings in this report. Undergoing the multiple panel genetic testing, the patient was hopeful to find a clear genetic cause for her disease; however, the patient remains unsure about the pathogenesis of her disease as the *PDGFRB* variant she carries is of unknown clinical significance, and she hopes that future functional analysis can prove or disprove the disease causality of her variant.

## Data availability statement

The original contributions presented in the study are included in the article/Supplementary material, further inquiries can be directed to the corresponding author.

## Ethics statement

Written informed consent was obtained from the individual (s) for the publication of any potentially identifiable images or data included in this article.

## Author contributions

JA performed data extraction, manuscript writing, and preparation of the figures. JY worked on data extraction, manuscript editing, and preparation of the table. MP worked on data extraction, manuscript editing, and preparation of the table. JF, JM, and KS conceived the study, edited the manuscript, and edited the figures and table. All authors contributed to the article and approved the submitted version.

## Conflict of interest

The authors declare that the research was conducted in the absence of any commercial or financial relationships that could be construed as a potential conflict of interest.

## Publisher’s note

All claims expressed in this article are solely those of the authors and do not necessarily represent those of their affiliated organizations, or those of the publisher, the editors and the reviewers. Any product that may be evaluated in this article, or claim that may be made by its manufacturer, is not guaranteed or endorsed by the publisher.

## Supplementary material

The Supplementary material for this article can be found online at: https://www.frontiersin.org/articles/10.3389/fneur.2023.1235909/full#supplementary-material
