# Supplementary material for: A case report of a patient with primary familial brain calcification with a PDGFRB genetic variant
Source: Front Neurol. 2023 Sep 14;14:1235909. doi: 10.3389/fneur.2023.1235909 (PMC10538541; doi:10.3389/fneur.2023.1235909)
Supplement: Supplementary file 1 [file Data_Sheet_1.docx]

**Detailed methods for genetic sequencing:**

Three gene panels were used to screen for genetic variants in a total of 70 genes implicated in neurodegeneration. The 1^st^ panel is the “Invitae Hereditary Parkinson Disease and Parkinsonism Panel with Add on Preliminary-evidence Genes”, test code 03352 (https://www.invitae.com/en/providers/test-catalog/test-03352). Genes included in this panel were the following: ATP13A2, ATP7B, CHCHD2, CSF1R, DCTN1, DNAJC6, FBXO7, GBA, GCH1, LRRK2, PARK7, PDE8B, PINK1, PLA2G6, PRKN, PRKRA, RAB39B, SLC6A3, SNCA, SPR, SYNJ1, TH, TMEM230, VPS13C, VPS35, XPR1, and the add-on preliminary-evidence genes: MAPT, PODXL, and UCHL1.

The 2^nd^ panel is the “Invitae Hereditary Amyotrophic Lateral Sclerosis, Frontotemporal Dementia and Alzheimer Disease Panel with Add-on Preliminary-evidence Genes”, test code 03502 (https://www.invitae.com/en/providers/test-catalog/test-03502). Genes included in this panel were the following: ALS2, ANG, ANXA11, APP, CHCHD10, CHMP2B, DCTN1, ERBB4, FUS, GRN, HEXA, HNRNPA2B1, ITM2B, KIF5A, MAPT, OPTN, PFN1, PRNP, PSEN1, PSEN2, SETX, SNCA, SOD1, SORL1, SPG11, SQSTM1, TARDBP, TBK1, TFG, TREM2, UBQLN2, VAPB, VCP, and the add-on preliminary-evidence genes: ATP13A2, DDHD1, ERLIN1, FIG4, LRRK2, MATR3, NEFH, SIGMAR1, and TIA1. In summary, the Invitae sequence analysis in these two panels covers clinically important regions of each gene as determined by Invitae, including coding exons and 10 to 20 base pairs of adjacent intronic sequence on either side of the coding exons. In addition, the analysis covers select non-coding variants, and Invitae’s deletion/duplication analysis determines copy number at a single exon resolution at virtually all targeted exons.

The 3^rd^ panel is the “PreventionGenetics panel for exome sequencing and CNV detection: Idiopathic Basal Ganglia Calcification Panel”, test code 10171 (https://www.preventiongenetics.com/testInfo?val=Idiopathic-Basal-Ganglia-Calcification-Panel). Genes included in this panel were the following: SLC20A2, PDGFB, PDGFRB and XPR1. In summary and according to the manufacturers report, sequence analysis is performed using Next-Gen sequencing (NGS) with additional Sanger sequencing as necessary. This panel provides 100% coverage of all coding exons of the genes plus 10 bases of flanking noncoding DNA in all available transcripts along with other non-coding regions in which pathogenic variants have been identified at PreventionGenetics or reported elsewhere. Copy number variants (CNVs) are also detected from NGS data and all CNVs are confirmed using another technology such as microarray-based comparative genomic hybridization (aCGH), Multiplex Ligation-depending Probe Amplification (MLPA), or polymerase chain reaction (PCR) before they are reported.
